# Effects of aquatic exercises on physical fitness and quality of life in postmenopausal women: an updated systematic review and meta-analysis

**DOI:** 10.3389/fpubh.2023.1126126

**Published:** 2023-06-08

**Authors:** Wen-Sheng Zhou, Su-Jie Mao, Shi-Kun Zhang, Hong Xu, Wei-Lu Li

**Affiliations:** ^1^College of Physical Education, Jiangsu Second Normal University, Nanjing, China; ^2^Nanjing Sport Institute, Nanjing, China; ^3^Department of Police Physical Education, Jiangsu Police Institute, Nanjing, China; ^4^Department of Sport and Health Science, College of Natural Science, Sangmyung University, Seoul, South Korea; ^5^Nanjing Zhong-Yang Road Primary School, Nanjing, China

**Keywords:** hydrotherapy, head-out water exercise, older women, physical performance, muscle strength, agility

## Abstract

**Objective:**

In the present systematic review and meta-analysis, we aimed to evaluate and update the effects of aquatic exercise on physical fitness and quality of life (QoL) in postmenopausal women.

**Methods:**

The databases Cochrane Library, PubMed, Web of Science, and MEDLINE were searched for randomized controlled trials (RCTs) on the topic from inception to July 2022. The GetData software was used to extract data from the published images. RevMan5.4 software was used for statistical analysis. Data are expressed as standardized mean difference (SMD) with 95% confidence intervals (CI). I^2^ index was employed for heterogeneity. Egger's test was used to assess publication bias. We evaluated the methodological quality of included studies using the Physiotherapy Evidence Database scale.

**Results:**

We included 594 participants in 16 RCTs (19 comparison groups). The results indicated that aquatic exercise can significantly improve lower limb strength (LLS), upper limb strength (ULS), agility, flexibility, and overall QoL. No significant effects were found on aerobic capacity. Subgroup-analysis results indicated that aquatic exercise only significantly improved LLS, ULS, agility, and flexibility in postmenopausal women < 65 years of age. However, aquatic exercise improves the overall QoL both in postmenopausal women < 65 years and ≥ 65 years. Aquatic resistance exercise significantly improves LLS, ULS, agility and flexibility. In addition, aquatic aerobic exercise can effectively increase LLS, and combined aquatic aerobic and resistance exercise can enhance the overall QoL.

**Conclusions:**

Aquatic exercise can effectively improve physical fitness and overall QoL in postmenopausal women, but has limited effects on aerobic capacity; thus, it is highly recommended in postmenopausal women.

## Introduction

With a rapidly aging of global population, the societies of many countries are gradually becoming aged (1, 2). A decline in muscle strength, cardiorespiratory fitness, mobility, and flexibility usually accompany the aging process (3–5). The body functional degeneration is more pronounced in older (6) and postmenopausal women (7). Postmenopausal women experience reduced muscle mass, muscle strength, and neuromuscular function due to ovarian degeneration and decreased secretion of estrogen, which in turn further exacerbates the degeneration of the ability to perform daily activities (8–10) and increases the risk of falling (7, 11). About 30% of people over 65 years fall at least once a year (12–14). Daily physical activity and motivation to participate in exercise are limited due to fear of falls and fractures, leading to a sedentary lifestyle and decreased quality of life (QoL) (15–19).

Exercise is a great means to improve physical fitness and emotional and mental health (20, 21). For persons with poor balance, fear of falling, joint pain, and weak muscle strength, aquatic exercise is a better alternative (22–24). Water buoyancy reduces joint load by 50–90%, especially good for people with decreased lower limb strength (LLS), obesity, and joint pain (22, 25). In older adults, aquatic resistance exercise increases muscle mass and strength and reduces the risk of falls (26, 27). Hydrostatic pressure increases blood circulation in the lower limbs (24). However, no consistent opinions have been reached regarding the efficacy of aquatic exercise on physical fitness and QoL in postmenopausal women. According to several authors, aquatic exercise can significantly improve LLS (1, 28–34), while Dong-Hyun et al. (35) found limited improvement in LLS (35). Ha et al. (1), Lopez et al. (32), and Perkins et al. (33) found that those who carried out aquatic exercise significantly achieved improved aerobic capacity compared to the control group (1, 32, 33), while Hafele, Alberton, Hafele et al. (31) had contrasting results (31). Dong-Hyun et al. (35) confirmed that aquatic aerobic exercise cannot significantly improve flexibility and there was no difference between the experimental group and the control group after aquatic exercise (35). Compared with before exercise in the study of Hafele, Alberton, Hafele et al. (31), 16 weeks of aquatic aerobic exercise and combined of aquatic aerobic and resistance exercise did not improve agility, and there was no difference between groups for agility (31). In a systematic review and meta-analysis, Saquetto et al. (36), confirmed that aquatic exercise can significantly improve LLS, flexibility, agility, and aerobic capacity (36). However, studies are lacking for the arrival at a consensus on the issue. In addition, the different types of exercises (aquatic aerobic, resistance, and multicomponent exercise) were not taken into account in most studies, which may result in different benefits from different exercise types. Furthermore, considering the different menopausal ages (37, 38), studies on the different physical fitness benefits were needed to analyze specifically from participating in aquatic exercise between young and older postmenopausal women. In terms of QoL, Hafele et al. (39) found that 16 weeks of aquatic aerobic exercise and combined aquatic aerobic and resistance exercises can significantly improve the overall QoL in postmenopausal women (39). Silva et al. (34) found that aquatic aerobic exercise significantly improved overall QoL compared with pre-exercise, unlike combined aerobic and resistance exercises (34). Therefore, taking into account the influences of exercise types and ages of participants, the present study systematically evaluated and updated the effects of aquatic exercise on physical fitness and overall QoL in postmenopausal women.

## Methods

The present study strictly followed the Preferred Reporting Items for Systematic Reviews and Meta-Analyses statement (40).

### Search strategy

We systematically searched the databases (Cochrane Library, PubMed, Web of Science, and MEDLINE) for randomized controlled trials (RCTs) using the following search terms: (aquatic exercise OR water-based exercise OR water exercise OR head-out water exercise) AND (functional fitness OR physical fitness OR physical capacity OR agility OR flexibility OR cardiorespiratory fitness OR aerobic capacity OR strength endurance OR strength OR quality of life) AND (postmenopausal women OR old women OR older women). All search terms were required to appear in the title or abstract. We also reviewed the reference list of the included literature. The Search was limited to database inception until July 2022. Two researchers (WSZ and SJM) independently completed the databases searching.

### Eligibility criteria

(i) RCTs; (ii) with exercise intervention as aquatic exercise or head-out water-based exercise; the control group did not participate in exercise. (iii) with participants being physiological postmenopausal women or women aged > 55 years (41–44); and (iv) with outcomes including physical fitness indicators and the overall QoL.

### Study selection and data extraction

Two researchers (W-SZ and S-JM) independently conducted the selection of titles and abstracts from each database. The full text was obtained if the researchers deemed one study eligible. Two researchers (W-SZ and S-JM) independently extracted the study characteristics from eligible articles, including authors, publication year, age, sample size, exercise interventions, and primary outcomes. The corresponding authors of these studies were contacted in case of missing data. We deleted articles whose authors could not be reached or could not provide the data. The GetData software was used for extracting data if the results were presented as figures in the included articles (45, 46). Two researchers (W-SZ and S-JM) independently completed the data extraction and review. A third researcher (S-KZ) was invited and a consensus was reached at in case of discrepancies.

### Quality assessment

The Physiotherapy Evidence Database (PEDro) scale was used to assess the methodological quality of the included articles. The PEDro scale is based on 11 items, including eligibility criteria (not contribute to the total score), random allocation, concealed allocation, similarity baseline, subject blinding, therapist blinding, assessor blinding, >85% retention, intention-to-treat, between-group comparisons, and point and variability measures. Each study was assessed as “yes” (1 point) or “no” (0 points), with a maximum total score of 10. A study is considered to be of very good quality if it has a score of 9 or 10, while a score of 6 to 8 indicates good quality, a score of 4 or 5 indicates moderate quality, and a score of 0 to 3 indicates poor quality (47, 48). Two researchers (S-KZ and HX) independently performed the quality assessment, and a third researcher (W-SZ) was invited and a consensus was reached at if there was any discrepancy.

### Statistical analysis

Data analysis was performed using the Cochrane Collaboration Review Manager (RevMan, version 5.4, Copenhagen, Denmark) software. Standardized mean difference (SMD) was employed if there were different outcome measures (49). I^2^ index was used to test statistic heterogeneity. An I^2^ >50% indicated high heterogeneity, and a random-effect model was applied (50). Sensitivity analysis was done by deleting studies one after the other (51). Egger's regression test was used to assess publication bias (52). The statistical significance level was set at *p* < 0.05.

## Results

### Search results

**Figure 1:**
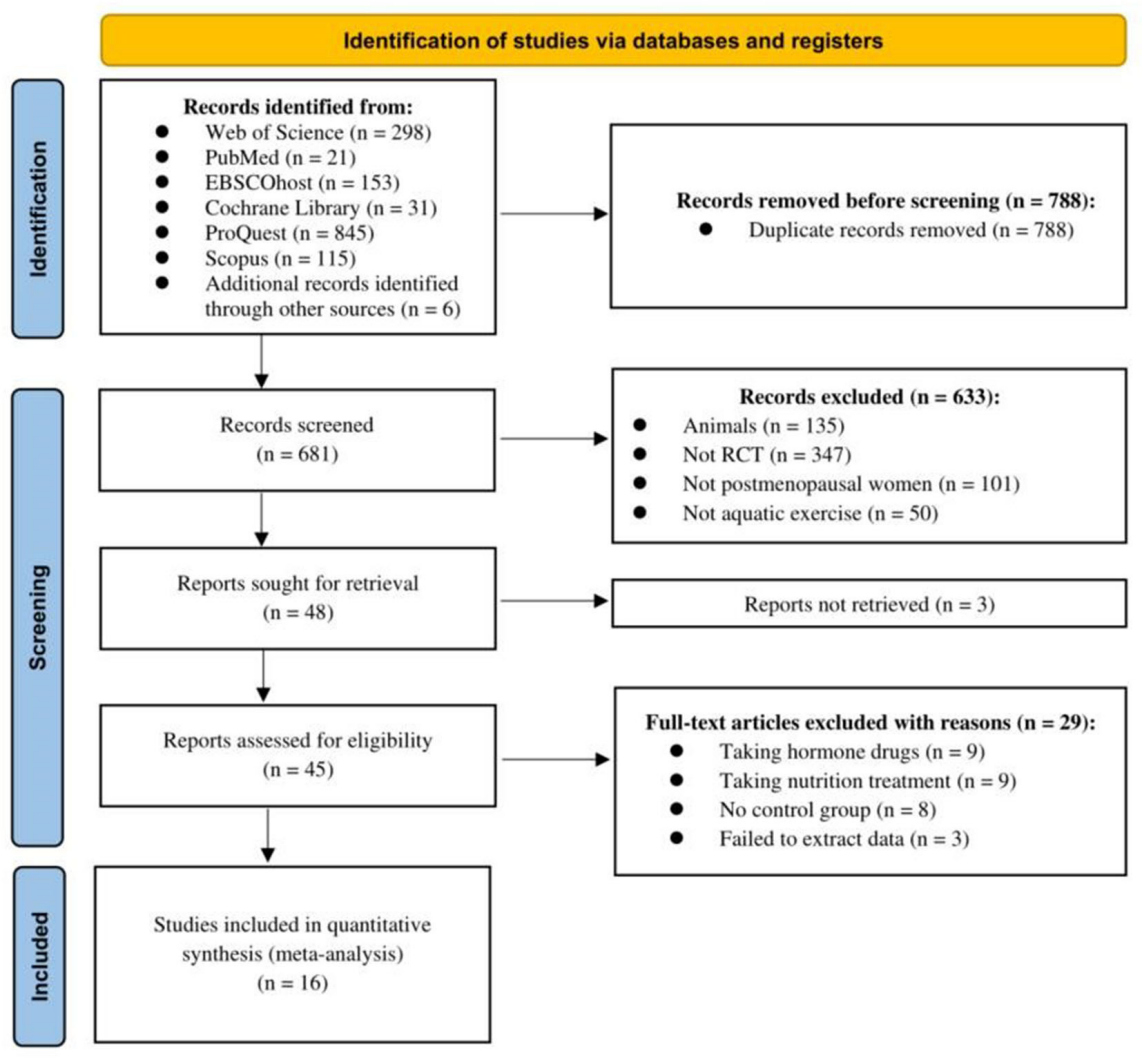
Flow diagram of search strategy.

Using the search strategy, 1,469 studies were retrieved, of which 1,421 studies were deleted because they were duplicates, animal studies, non-RCTs, or included non-postmenopausal women, and so on. Because the participants of 29 studies were on hormonotherapy, nutritional care, or the studies had no control group or failed to extract data, these studies were deleted. Sixteen RCTs (19 comparison groups) were finally included in the present study (Figure 1).

### Study characteristics

The 16 RCTs were published between 2006 and 2022 and involved 594 participants (320 received aquatic exercise interventions). The participants were aged from 54 to 74.9 years. Exercise duration was from 8 to 24 weeks, exercise frequency was from 2 to 5 times per week, and exercise time was from 30 to 60 min. Aquatic aerobic exercise (1, 28, 31, 32, 34, 35, 39), aquatic resistance exercise (29, 30, 53–55), and multicomponent exercise (aquatic aerobic and resistance exercises) (27, 31, 33, 34, 39, 56, 57) were the main types of exercises involved. The studies of Hafele, Alberton, Hafele, et al. (2022), Hafele, Alberton, Schaun, et al. (2022), and Silva et al. (2018) included both aquatic aerobic and multicomponent exercise (31, 34, 39). The participants compliance ranges from 73.6 to 100%. Each exercise session was supervised by the researchers. The characteristics of the included studies are summarized in Table 1.

**Table 1:** Characteristics of the included studies (*n* = 16).

**Authors, Year**	**Age**	**Sample size Compliance (AEG/CG)**	**AEG**	**Control**	**Water level, Temperature**	**Measured outcomes**				
						**Overall QoL**	**Muscle strength**	**Flexibility**	**Aerobic capacity**	**Agility**
Aboarrage et al. (28)	65 ± 7	25(15/10) (100%/100%)	20 min main aquatic exercise, 30 min × 3 times/week × 24 weeks	No report	xiphoid, 29°C		30-s CS			8-ft TUG
Araújo et al. (53)	54 ± 4	18(10/8) (88 ± 8%/89 ± 5%)	20 min low limbers resistance exercise, 45 min × 3 times/week × 8 weeks	Daily routine	xiphoid, 26–29°C					3-m TUG
Bento et al. (56)	65.8 ± 4.47	36(20/16) (no report)	20 min aerobic activities and 20 min lower limb strength exercises, 60 min × 3 times/week × 12 weeks	daily routine	xiphoid, 28–30°C					8-ft TUG
Bocalini et al. (29)	63.3 ± 1.09	35(25/10) (92.6%/50%)	45 min endurance-type exercise (arms and legs resistance exercises), 60 min × 3 times/week × 12 weeks	Daily routine	xiphoid, 29°C		30-s AC 30-s CS	SR		8-ft TUG
Bocalini et al. (30)	>62	45(27/18) (90%/90%)	45min endurance training, 60 min × 3 times/week × 12 weeks	Daily activities	no report	WHO-QoL	30-s AC 30-s CS	SR		8-ft TUG
Colado et al. (54)	54 ± 2.12	25(15/10) (>95%)	35–60 min resistance exercise, 35–60 min × 2–3 times/week × 24 weeks	Daily routine	no report			SR		
Dong-Hyun et al. (35)	72.2 ± 4.26	36(18/18) (no report)	40 min aquarobics exercise, 60 min × 3 times/week × 12 weeks	Daily routine	1.1-m, 28–29°C		30-s CS	SR		2.44-m TUG
Ha et al. (1)	74.9 ± 4.76	19(11/8) (no report)	40 min main exercise, 50 min × 3 times/week × 12 weeks	Daily routine	26–28°C		30-s CS	SR	6MWT	8-ft TUG
Hafele et al. (31)	66.15 ± 4.00	52(17/18/17) (100%/100%)	AE: 45 min aerobic exercise; ME: combined of aerobic and resistance training, 60 min × 3 times/week × 16 weeks	Aquatic therapeutic once A week	xiphoid-shoulders 32°C	WHO-QoL				
Hafele et al. (31)	66.15 ± 4.00	52(17/18/17) (100%/100%)	AE: 45 min aerobic exercise; ME: a combination of aerobic and resistance training, 60 min × 3 times/week × 16 weeks	Aquatic therapeutic once A week	xiphoid-shoulders 32°C		30-s CS	SR	6MWT	8-ft TUG
Lopez et al. (32)	74.4 ± 12.69	26(16/10) (73.6%)	30 min aerobic exercise, 50 min × 5 times/week × 12 weeks	Normal activities	no report		30-s AC 30-s CS	SR	6MWT	2.4-m TUG
Moreira et al. (57)	58.8 ± 6.4	108(64/44) (92.2%/93.2%)	30–40 min strength/power exercises and cardiorespiratory training, 50–60 min × 3 times/week × 24 weeks	No regular exercise	1.1–1.3-m 30–31°C			SR		3-m TUG
Perkins et al. (33)	57 (45–78)	38(26/12) (90%)	40 min aerobic routines, 60 min × 5 times/week × 17 weeks	No regular exercise	29.5°C		30-s CS	SR	6MWT	TUG
Sattar et al. (55)	54.9 ± 4.02	24(14/10) (100%)	30–40 min resistance exercise, 60 min × 3 times/week × 8 weeks	No regular exercise	28–30°C			SR		3-m TUG
Silva et al. (34)	65 ± 4	33(13/11/9) (88 ± 8%/89 ± 5%)	AE: aerobic exercise; ME: a combination of aerobic and resistance training, 2 times/week × 12 weeks	Non-periodized dance/gymnastics	no report	WHO-QoL	30-s CS		6MWT	8-ft TUG
Tsourlou et al. (27)	68.9 ± 4.62	22(12/10) (85.7%/100%)	45 min aerobic and resistance exercise, 60 min × 3 times/week × 24 weeks	Normal activities	0.9-m, 30°C			SR		3-m TUG

AEG, aquatic exercise group; CG, control group; AT, aerobic exercise; ME, multicomponent exercise; overall QoL, overall quality of life; CS, chair stand test; AC, arm curl test; SR, chair sit and reach test; 6MWT, 6 min walking test; TUG, timed up and go. WHO-QoL, The World Health Organization quality of life assessment.

### Summary of risk of bias

The ranges of the quality assessment scores of the included studies was from 3–6. Two studies received scores of 6 (good quality) (31, 39), 13 studies received scores of 4–5 (median quality) (1, 27–30, 32–35, 54–57), and 1 study received scores of 3 (poor quality) (53). The mean score was 4.8 (Table 2).

**Table 2:** Quality assessment of included studies (*n* = 16).

	**Included studies**	**1**	**2**	**3**	**4**	**5**	**6**	**7**	**8**	**9**	**10**	**11**	**Total score**
1	Aboarrage et al. (28)	1	1	0	1	0	0	0	1	0	1	1	5
2	Araújo et al. (53)	0	1	0	1	0	0	0	0	0	1	0	3
3	Bento et al. (56)	1	1	0	1	0	0	1	0	0	1	1	5
4	Bocalini et al. (29)	1	1	0	1	0	0	0	1	0	1	1	5
5	Bocalini et al. (30)	1	1	0	1	0	0	0	1	0	1	1	5
6	Colado et al. (54)	1	1	0	1	0	0	0	0	0	1	1	4
7	Dong-Hyun et al. (35)	1	1	0	1	0	0	0	1	0	1	1	5
8	Ha et al. (1)	0	1	0	1	0	0	0	1	0	1	1	5
9	Hafele et al. (31)	1	1	0	1	0	0	0	1	1	1	1	6
10	Hafele et al. (31)	1	1	0	1	0	0	0	1	1	1	1	6
11	Lopez et al. (32)	1	1	0	1	0	0	0	1	0	1	1	5
12	Moreira et al. (57)	1	1	0	1	0	0	0	1	0	1	0	4
13	Perkins et al. (33)	1	0	0	0	0	1	1	0	0	1	1	4
14	Sattar et al. (55)	0	1	0	1	0	0	0	1	0	1	1	5
15	Silva et al. (34)	1	1	1	0	0	0	1	0	0	1	1	5
16	Tsourlou et al. (27)	0	1	0	1	0	0	0	1	0	1	1	5

1, eligibility criteria (not contribute to the total score); 2, random allocation; 3, concealed allocation; 4, similarity baseline; 5, subject blinding; 6, therapist blinding; 7, assessor blinding; 8, >85% retention; 9, intention-to-treat; 10, between- group comparisons; 11, point and variability measures.

#### Effects of exercise on physical performance and quality of life

##### lower limbs strength-30-second chair stand test

**Figure 2:**
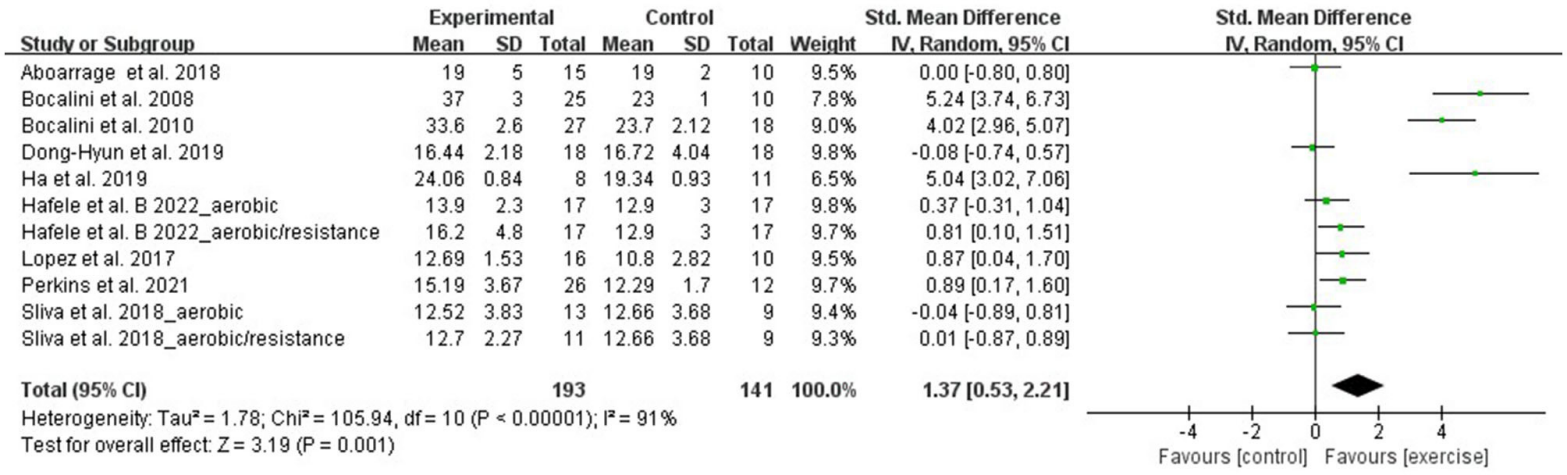
Forest plot of the effect of aquatic exercise on lower limbs strength (kg).

Using the 30-second chair stand test, LLS was evaluated by 11 RCTs that involved 334 participants. Due to the difference between the studies' assessments, the meta-analysis was performed with SMD. A random-effect model was applied for instances with a high heterogeneity (I^2^ = 91%, *p* < 0.00001). Sensitivity analysis results indicated that excluding any single study resulted in no significant effect on the total effect size. Meta-analysis results demonstrated that aquatic exercise can significantly increase LLS (SMD = 1.37, 95% CI: 0.53, 2.21, *p* = 0.001) (Figure 2).

Subgroup analysis showed that LLS was significantly increased in the age < 65 years (SMD = 3.33, 95% CI: 0.62, 6.03, *p* = 0.02), aquatic aerobic exercise (SMD = 0.81, 95% CI: 0.03, 1.59, *p* = 0.04), and Aquatic resistance exercise subgroups (SMD = 4.51, 95% CI: 3.33, 5.68, *p* < 0.00001). No significant effects were found in the age ≥ 65 years (SMD = 0.55, 95% CI: −0.04, 1.13, *p* = 0.07) and multicomponent exercise subgroups (SMD = 0.32, 95% CI: −0.23, 0.87, *p* = 0.26) when compared with the control group (Table 3).

**Table 3:** Subgroup results of aquatic exercise on physical performance and quality of life according to different age groups and exercise types.

**Outcomes**	**Group**	**Subgroup**	**N (AEG/CG)**	**SMD, 95% CI**	**(SMD) *p* value**	**I^2^ (%)**	**(I^2^) *p* value**
LLS	Age	Age < 65 years	78/40	3.33 [0.62, 6.03]	0.02	95.0	< 0.00001
		Age ≥ 65 years	115/101	0.55 [-0.04, 1.13]	0.07	75.0	0.0003
	Exercise type	Aerobic exercise	98/77	0.81 [0.03, 1.59]	0.04	81.0	< 0.00001
		Resistance exercise	52/28	4.51 [3.33, 5.68]	< 0.00001	42.0	0.19
		Multicomponent exercise	43/36	0.32 [-0.23, 0.87]	0.26	31.0	0.23
ULS	Age	Age < 65 years	52/28	2.44 [1.74, 3.15]	< 0.00001	22.0	0.26
		Age ≥ 65 years	16/10	0.58 [-0.23, 1.39]	0.16	Not applicable	Not applicable
	Exercise type	Aerobic exercise	16/10	0.58 [-0.23, 1.39]	0.16	Not applicable	Not applicable
		Resistance exercise	52/28	2.44 [1.74, 3.15]	< 0.00001	22.0	0.26
Agility	Age	Age < 65 years	166/102	−0.98 [-1.51,−0.44]	0.0003	71.0	0.004
		Age ≥ 65 years	148/125	−0.49 [-1.07, 0.10]	0.10	80.0	< 0.00001
	Exercise type	Aerobic exercise	98/75	−0.25 [-0.79, 0.30]	0.38	65.0	0.01
		Resistance exercise	76/46	−1.35 [-1.87,−0.83]	< 0.00001	35.0	0.20
		Multicomponent exercise	140/106	−0.68 [-1.46, 0.10]	0.09	85.0	< 0.00001
Aerobic capacity	Age	Age < 65 years	26/12	0.25 [-0.43, 0.94]	0.47	Not applicable	Not applicable
		Age ≥ 65 years	85/70	0.55 [-0.20, 1.08]	0.15	78.0	0.0003
	Exercise type	Aerobic exercise	83/56	0.68 [-0.23, 1.59]	0.14	82.0	0.0001
		Multicomponent exercise	28/26	0.15 [-0.38, 0.69]	0.57	0	0.93
Flexibility	Age	Age < 65 years	171/104	1.38 [0.37, 2.39]	0.008	91.0	< 0.00001
		Age ≥ 65 years	91/80	0.52 [-0.07, 1.10]	0.08	70.0	0.005
	Exercise type	Aerobic exercise	76/63	0.51 [-0.22, 1.24]	0.17	76.0	0.002
		Resistance exercise	67/38	2.49 [0.14, 4.84]	0.04	95.0	< 0.00001
		Multicomponent exercise	119/83	0.57 [-0.02, 1.17]	0.06	71.0	0.02
Overall QoL	Age	Age < 65 years	27/18	2.98 [2.10, 3.85]	< 0.00001	Not applicable	Not applicable
		Age ≥ 65 years	45/38	0.54 [0.10, 0.99]	0.02	0	0.76
	Exercise type	Aerobic exercise	24/19	0.36 [-0.25, 0.97]	0.25	0	0.53
		Resistance exercise	27/18	2.98 [2.10, 3.85]	< 0.00001	Not applicable	Not applicable
		Multicomponent exercise	21/19	0.75 [0.11, 1.40]	0.02	0	0.92

AEG, aquatic exercise group; CG, control group; LLS, lower limbs strength; ULS, upper limbs strength; Overall QoL, overall quality of life; SMD, standard mean difference; CI, confidence interval.

#### Upper limbs strength-arm curl test

**Figure 3:**
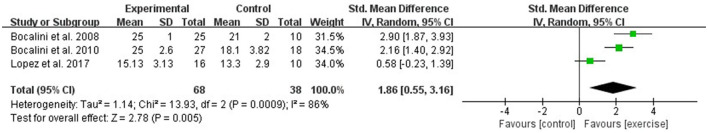
Forest plot of the effect of aquatic exercise on upper limbs strength (kg).

Using the arm curl test, upper limbs strength (ULS) was evaluated by 3 RCTs that involved 106 participants. A meta-analysis was performed with SMD. A random-effect model was applied in instances with a high heterogeneity (I^2^ = 86%, *p* = 0.0009). After removing the study of Lopez et al. (32), sensitivity analysis results indicated that the heterogeneity was lower (I^2^ = 22%, *p* = 0.26). However, the total effect size did not change significantly. The meta-analysis results demonstrated that aquatic exercise can significantly increase ULS (SMD = 1.86, 95% CI: 0.55, 3.16, *p* = 0.005) when compared with the control group (Figure 3).

The RCTs of age < 65 years and aquatic resistance exercise subgroups were from the studies of Bocalini et al. (29) and Bocalini et al. (30). Subgroup results showed that ULS had significantly increased in the above two subgroups (SMD = 2.44, 95% CI: 1.74, 3.15, *p* < 0.00001). The RCTs of age ≥ 65 years were from the study of Lopez et al. (32) and no significant effects were found in the age ≥ 65 years subgroup (SMD = 0.58, 95% CI: −0.23, 1.39, *p* = 0.16) (Table 3).

#### Agility-timed up and go test

**Figure 4:**
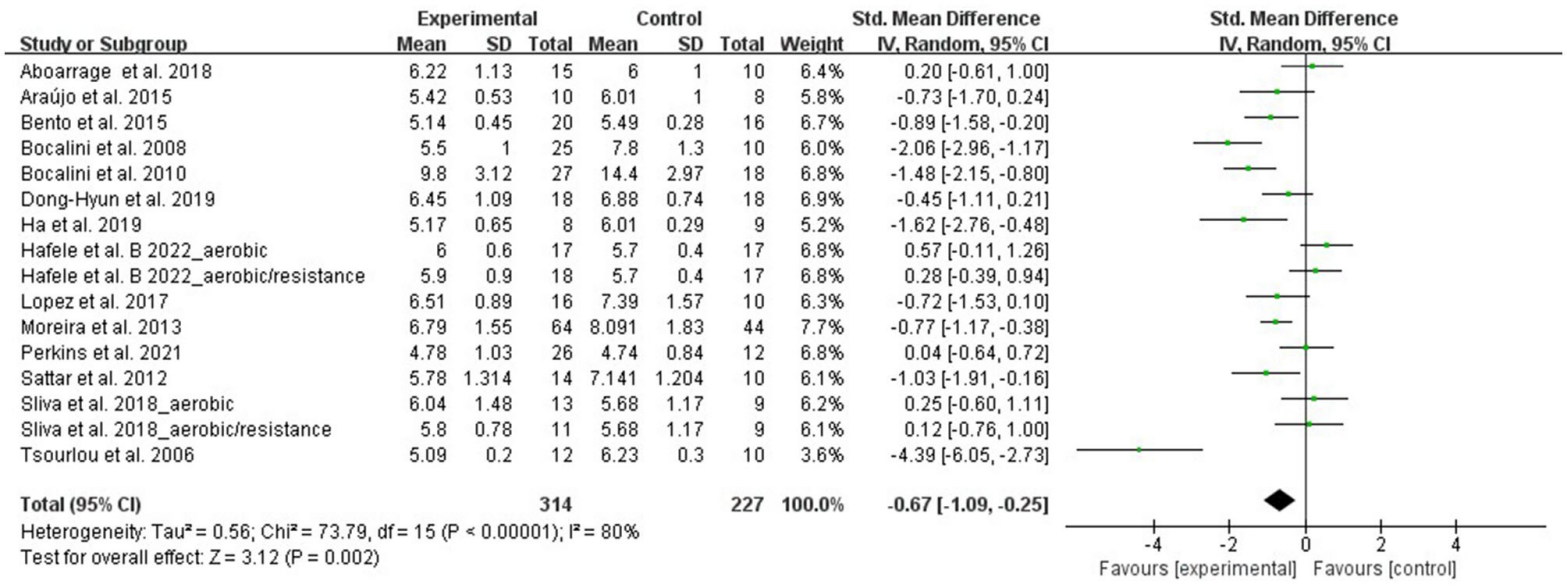
Forest plot of the effect of aquatic exercise on agility (sec).

Using the timed up and go test, agility was evaluated by 16 RCTs that involved 541 participants. Due to the difference between the studies' assessments, the meta-analysis was performed with SMD. A random-effect model was applied in instances with a high heterogeneity (I^2^ = 80%, *p* < 0.00001). Sensitivity analysis results indicated that excluding any single study resulted in no significant effect on the total effect size. The meta-analysis results demonstrated that aquatic exercise can significantly improve agility (SMD = −0.67, 95% CI: −1.09, −0.25, *p* = 0.002) when compared with the control group (Figure 4).

Subgroup analysis results showed that agility was significantly improved in the age < 65 years (SMD = −0.98, 95% CI: −1.51, −0.44, *p* = 0.0003) and aquatic resistance exercise subgroups (SMD = −1.35, 95% CI: −1.87, −0.83, *p* < 0.00001). No significant effects were found in the age ≥ 65 years (SMD = −0.49, 95% CI: −1.07, 0.10, *p* = 0.10), aquatic aerobic exercise (SMD = −0.25, 95% CI: −0.79, 0.30, *p* = 0.38), and multicomponent exercise subgroups (SMD = −0.68, 95% CI: −1.46, 0.10, *p* = 0.09) (Table 3).

#### Aerobic capacity- 6-min walking test

**Figure 5:**
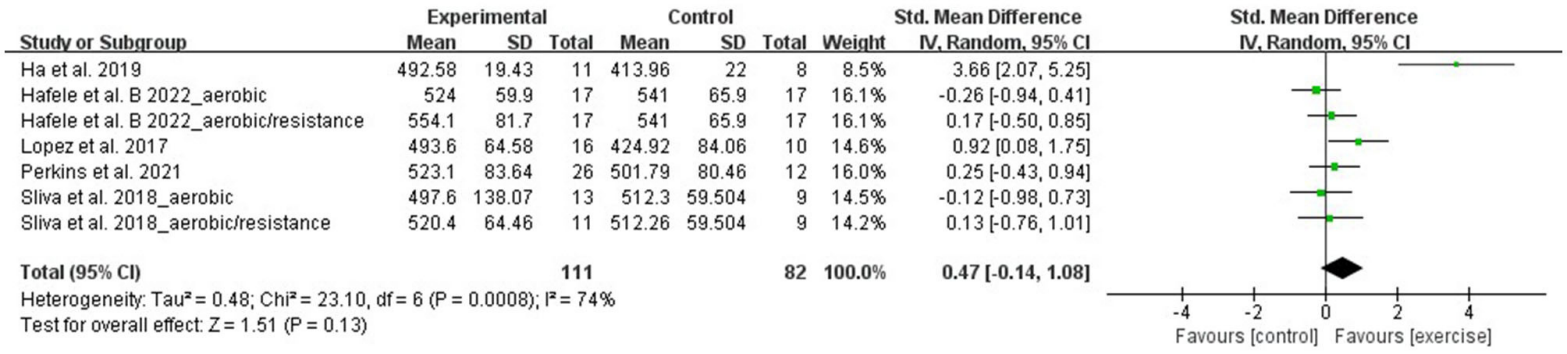
Forest plot of the effect of aquatic exercise on aerobic capacity (m).

Using the 6-minute walking test (6MWT), aerobic capacity was evaluated by 7 RCTs that involved 193 participants. Due to the difference between the studies' assessments, the meta-analysis was performed with SMD. A random-effect model was applied for instances with a high heterogeneity (I^2^ = 74%, *p* = 0.0008). Sensitivity analysis results indicated that the heterogeneity was lower (I^2^ = 3%, *p* = 0.39) after removing the study of Ha et al. (1). However, the total effect size did not change significantly. The meta-analysis results demonstrated that aquatic exercise does not significantly improve aerobic capacity (SMD = 0.47, 95% CI: −0.14, 1.08, *p* = 0.13) when compared with the control group (Figure 5).

Subgroup results showed that aerobic capacity was not significantly improved in the age < 65 years (SMD = 0.25, 95% CI: −0.43, 0.94, *p* = 0.47), age ≥ 65 years (SMD = 0.55, 95% CI: −0.20, 1.29, *p* = 0.15), aquatic aerobic exercise (SMD = 0.68, 95% CI: −0.23, 1.59, *p* = 0.14), and multicomponent exercise subgroups (SMD = 0.15, 95% CI: −0.38, 0.69, *p* = 0.57) (Table 3).

#### Flexibility-chair sit and reach test

**Figure 6:**
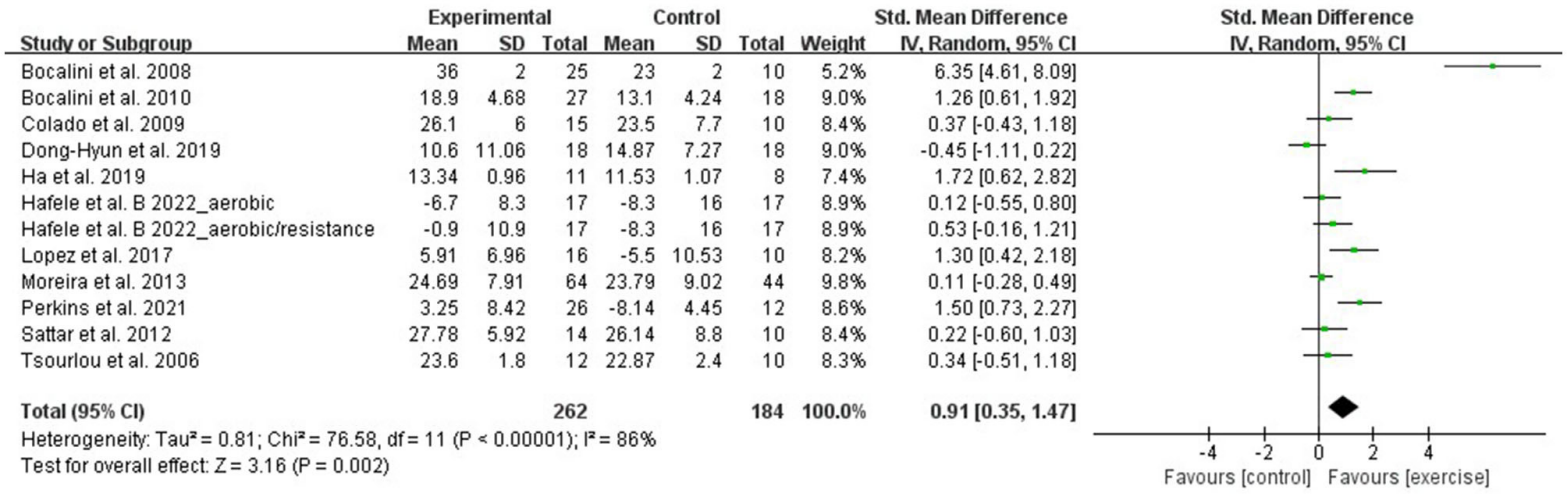
Forest plot of the effect of aquatic exercise on flexibility (cm).

Using the chair sit and reach test, flexibility was evaluated by 12 RCTs that involved 446 participants. Due to the difference between the studies' assessments, the meta-analysis was performed with SMD. A random-effect model was applied for instances with a high heterogeneity (I^2^ = 86%, *p* < 0.00001). Sensitivity analysis results indicated that excluding any single study resulted in no significant effect on the total effect size. The meta-analysis results demonstrated that aquatic exercise can significantly improve flexibility (SMD = 0.91, 95% CI: 0.35, 1.47, *p* = 0.002) when compared with the control group (Figure 6).

Subgroup results showed that flexibility was significantly improved in the age < 65 years (SMD = 1.38, 95% CI: 0.37, 2.39, *p* = 0.008) and aquatic resistance exercise subgroups (SMD = 2.49, 95% CI: 0.14, 4.84, *p* = 0.04). No significant effects were found in the age ≥ 65 years (SMD = 0.52, 95% CI:−0.07, 1.10, *p* = 0.08), aquatic aerobic exercise (SMD = 0.51, 95% CI: −0.22, 1.24, *p* = 0.17), and multicomponent exercise subgroups (SMD = 0.57, 95% CI: −0.02, 1.17, *p* = 0.06) (Table 3).

#### Overall quality of life

**Figure 7:**
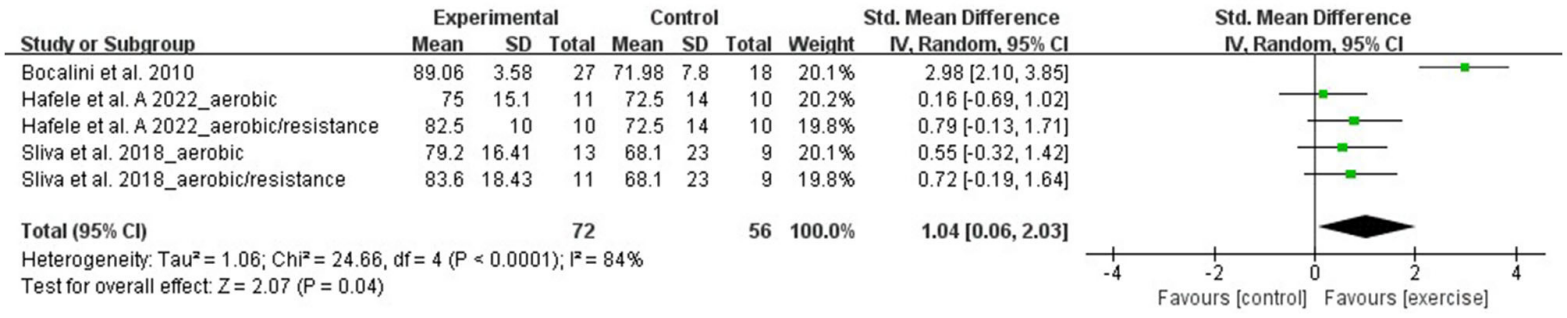
Forest plot of the effect of aquatic exercise on overall quality of life.

Five RCTs that involved 128 participants evaluated overall QoL using the World Health Organization quality of life assessment. Due to the difference between the studies' assessments, the meta-analysis was performed with SMD. A random-effect model was applied for instances with a high heterogeneity (I^2^ = 84%, *p* < 0.0001). Sensitivity analysis results indicated that the heterogeneity was lower (I^2^ = 0, *p* = 0.76) after removing the study of Bocalini et al. (30). However, the total effect size did not change significantly. The meta-analysis results demonstrated that when compared with the control group, aquatic exercise can significantly improve overall QoL (SMD = 1.04, 95% CI: 0.06, 2.03, *p* = 0.04) (Figure 7).

The RCTs of age < 65 years and aquatic resistance exercise subgroups were from the study of Bocalini et al. (30). Subgroup analysis results showed that overall QoL was significantly improved in the age < 65 years, aquatic resistance exercise (SMD = 2.98, 95% CI: 2.10, 3.85, *p* < 0.00001), age ≥ 65 years (SMD = 0.54, 95% CI: 0.10, 0.99, *p* =0.02), and multicomponent exercise subgroups (SMD = 0.75, 95% CI: 0.11, 1.40, *p* =0.02). No significant effects were found in the aquatic aerobic exercise subgroup (SMD = 0.36, 95% CI: −0.25, 0.97, *p* = 0.25) (Table 3).

### Publication bias

Egger test revealed that there was a relatively higher level of publication bias in aerobic capacity (*t* = −2.67, *p* = 0.045). There was no obvious publication bias in LLS (*t* = −1.38, *p* = 0.201), ULS (*t* = −0.02, *p* = 0.984), agility (*t* = −0.24, *p* = 0.815), flexibility (*t* = 0.21, *p* = 0.838), and overall QoL (*t* = −1.76, *p* = 0.177) (Supplementary material).

## Discussion

The present systematic review and meta-analysis demonstrated that aquatic exercise can effectively improve LLS, ULS, agility, flexibility, and overall QoL in postmenopausal women, but has limited effects on aerobic capacity. Aquatic exercise can only significantly improve LLS, ULS, agility, and flexibility in postmenopausal women < 65 years old. There was a significant improvement in overall QoL for both < 65 and ≥ 65 years old. As per our findings, aquatic resistance exercise is the best option for postmenopausal women to improve physical fitness and QoL.

### Lower- and upper- limbs strength

A decline in muscle mass and balance ability in the older adults commonly accompanies the aging process. This leads to limited mobility and loss of independent living ability, which greatly reduces the quality of life of the older adults (58, 59). It is particularly important to maintain LLS in the older adults. Past studies have indicated that LLS is the basic fitness of balance ability and an important factor in fall prevention in the older adults (60). The present study demonstrated that aquatic exercise significantly increases LLS (SMD = 1.37, *p* = 0.001, *n* = 334) and ULS (SMD = 1.86, *p* = 0.005, *n* = 106). The present findings conform well to those of Saquetto et al. (36), who observed that aquatic exercise significantly increases the muscle strength of knee extension (SMD = 3.34, *p* = 0.004, *n* = 216), knee flexion (SMD = 2.51, *p* = 0.007, *n* = 82), and arm curl (SMD = 6.78, *p* = 0.0001, *n* = 80). In the present review, 11 studies analyzed LLS, and 3 studies analyzed ULS, and the sample size of the included studies was larger. Therefore, our findings further updated the results of the previous study. The results of aquatic exercise efficacy in improving upper and lower extremity muscle strength were confirmed. According to many studies, resistance training can significantly improve muscle strength (61, 62). Resistance exercise in water is a great way for increasing strength, especially for ULS. Most of the studies included showed that the depth of the water reaches the xiphoid, which is beneficial for applying ULS exercise and increasing ULS (63). In addition, walking or jumping in the water is challenged by the drag and resistance of the water, thereby improving lower body muscle strength. The physiological mechanism of aquatic exercise to improve muscle strength mainly due to the improvement of neuromuscular system function (64). The trend of muscle strength decline was reversed (64). Previous studies have suggested that degenerated skeletal muscle recruitment patterns and functions are the main cause of decreased muscle strength (65, 66). Resistance training increases muscle strength by improving neuromuscular recruitment and muscle contraction (66, 67). Moreover, it may also be related to increased muscle mass, which is the main cause of increased muscle strength (68). Previous research confirmed that aquatic exercise significantly increases skeletal muscle mass (69, 70).

It is worth noting that aquatic exercise significantly improves LLS in postmenopausal women < 65 years old (SMD = 3.33, *p* = 0.02), but no efficacy was found in the ≥ 65 years subgroup. One possible explanation was that higher age-related muscle weakness, poor joint mobility, and poor balance limited body movement in a water environment lead to insufficient exercise intensity (71). Regarding the exercise types, both aquatic aerobic exercise (SMD = 0.81, *p* = 0.04) and resistance exercise (SMD = 4.51, *p* < 0.00001) significantly improved LLS. Aquatic resistance exercises induced greater magnitudes of improvement.

Regarding the ULS index, the same two studies were included in the age < 65 years and aquatic resistance exercise subgroups (29, 30). ULS was significantly improved (SMD = 2.44, *p* < 0.00001), but no efficacy was found in the age ≥ 65 years and aerobic exercise subgroups (SMD = 0.58, *p* = 0.16). Data for the age ≥ 65 years and aerobic exercise subgroups are from the same study (32). Therefore, the results of these two subgroups should be interpreted and applied with caution, and more studies are needed to be included for a more comprehensive interpretation in the future.

### Agility

As ages increase, it becomes increasingly challenging for the elder to move quickly and change direction (72, 73). Decreased agility is a key factor in predicting risk for recurrent falls (74). Exercise training is an important way to maintain and improve agility (75). In the present study, agility was significantly improved in the aquatic exercise group compared with the no exercise group (SMD = −0.67, *p* = 0.002). Our study results were in accordance with the meta-analysis of Saquetto et al. (36), in which 165 participants were included, and agility was significantly increased (SMD = −2.13, *p* = 0.05). Compared with the study of Saquetto et al. (36), our study has a superiority in including more studies (16 RCTs) and a larger sample size. The effect of improved neuromuscular function on increased muscle strength of the upper and lower limbs may have been responsible for the positive results (76, 77). Agility is the comprehensive embodiment of strength, speed, balance, and coordination, moreover, strength is the foundation of agility (78), and is directly associated with neuromuscular function status (79). In the present study, the overall results showed a significant increase in LLS of 1.37 kg and ULS of 1.86 kg. The results of subgroup showed that agility was improved only in the subgroup with age < 65 years (SMD = −0.98, *p* = 0.0003). Moreover, only postmenopausal women aged < 65 years showed significant improvement in LLS (SMD = 3.33, *p* = 0.02) and ULS (SMD = 2.44, *p* < 0.00001), further emphasizing the importance of strength in improving agility (79, 80). This may also be related to the improvement of joint range of motion. Previous studies have pointed out that agility and flexibility have a significant positive correlation (81), namely the better the flexibility performance, the shorter the agility test time. The present study also indicated that aquatic exercises can significantly improve the flexibility of lower limbs, and thereunto, only the flexibility of the subgroup aged < 65 years was significantly improved (SMD = 1.38, *p* = 0.008). Therefore, it is believed that the improvement of flexibility may be one of the possible reasons for the improvement of agility. Among the subgroups, agility was found to be improved in the age < 65 years subgroup, emphasizing that changes in agility improved by aquatic exercise were associated with age, precisely a relatively young age can contribute to better effects. Furthermore, subgroup analysis indicated that only aquatic resistance exercises can improve agility, reminding us that the importance and particularity of aquatic resistance exercises should be significantly considered when designing aquatic exercise programs in the future.

### Aerobic capacity

6MWT, as an important index that assesses the aerobic capacity, was adopted in the included studies. 6MWT was a submaximal exercise ability test for the middle-aged and the old adults (82, 83). The present study demonstrated that aquatic exercises cannot significantly improve aerobic capacity in postmenopausal women. In addition, subgroup analysis showed that the aerobic capacity was not improved in the aged < 65 years (*n* = 38), aged ≥ 65 years (*n* = 155), aerobic exercise (*n* = 139), and multicomponent exercise subgroups (*n* = 54). Our findings are consistent with those of the study of Ha et al. (1), who included participants performing aquatic aerobic exercises 3 times a week for a 12-week duration (1). They found that 6MWT was not improved. No significant improvement of 6MWT was found in the study of Perkins et al. (2021) with 60 min of aerobic exercises 5 times a week for a 17-week duration (33). The controversial findings were possibly associated with the low impact when performing aquatic exercises, which was caused by buoyancy and reduced the muscle loads. Although the resistance to water caused the muscle to produce a contractile load, it may not be sufficient to induce a large cardiopulmonary response and therefore did not produce a better adaptive increase in cardiopulmonary function. Furthermore, it may also be related to the lower heart rate level during aquatic exercises. To our knowledge, the reduction in heart rate was mainly due to hydrostatic pressure. Hydrostatic pressure increases venous return and decreases peripheral blood volume. As a result, end-diastolic volume and stroke volume increase, thereby reducing the heart rate (84, 85), accompanied by increased vagal and parasympathetic activity and decreased sympathetic activity caused by atrial and arterial baroreflex mechanisms (86, 87). This results in a lower cardiopulmonary load without an adaptive increase in cardiopulmonary function. We mainly adopted 6MWT to assess the aerobic capacity, and there were also other studies using the VO_2max_ index. The study of Saquetto et al. (36) using VO_2max_ index included 4 articles and the results demonstrated that aquatic exercises could increase VO_2max_ by 4.12 ml/kg, which was inconsistent with the results of our study. Therefore, there was still some controversy about the results of aquatic exercise on improving the aerobic capacity level, and we should be especially cautious when interpreting and applying these results. Our study indicated that there was a relatively higher level of publication bias in aerobic capacity. It may be mainly related to the small number of the included articles. It is well known that participating in exercise for a period of time is helpful to improve aerobic capacity (88), but in the end, it shows negative results in the present study. It is also may be that the research articles with positive results have not been published or published in non-English journals.

### Flexibility

Decreased flexibility is associated with the development of musculoskeletal disorders, progressive disability (89), and an increased risk of falls in middle-aged and old adults (90). Exercise is a favorable way to maintain and improve flexibility (91). The present study demonstrated that aquatic exercises can improve lower limb flexibility in postmenopausal women (SMD = 0.91, *p* = 0.002). Our study results were consistent with the study of Saquetto et al. (36), including only 3 RCTs vs. 12 RCTs in our study, thereby confirming the effectiveness of aquatic exercises on improving lower limb flexibility. Previous studies have shown that exercises are efficacious in improving flexibility. Aquatic exercises, as an intervention method in our study, can make people more relaxed and the action more stretched. Moreover, the buoyancy of water can reduce the fear of falling in middle-aged and old postmenopausal women, and higher water temperature had the effect of hot compress and massage, which further reduced the stiffness of tissues and muscles around joints (92), thus bringing better advantages to the improvement of joint mobility. In addition, our study has confirmed the effectiveness of aquatic exercise on improving muscle strength, especially the increased muscle strength of the lower limbs leading to more stable joints. Under the hydrostatic pressure of water, the blood circulation is better, the blood flow around the joint is more, and the metabolic wastes are recovered and disposed of, thus improving the range of motion of the joint. The subgroup analysis results of the study showed that the flexibility of the aged < 65 years (SMD = 1.38, *p* = 0.008) and the resistance exercises subgroups (SMD = 2.49, *p* = 0.04) was significantly increased, while the aged ≥ 65 years, aerobic exercises, and combination exercises subgroups were not significantly improved. It followed then that aquatic exercise can improve the flexibility of postmenopausal women aged < 65 years, and resistance exercise had a better effect on improving flexibility. Due to the importance of flexibility to the ability of voluntary physical activity and fall prevention, older adults should maintain a certain range of joint motion (93).

### Quality of life

The decline of physical function associated with aging influenced the ability of daily independent living of older adults. Meanwhile, they had various chronic diseases and emotional burdens, which affect the overall QoL (16, 94, 95). The present study demonstrated that aquatic exercises can significantly improve the overall QoL in postmenopausal women (SMD = 1.04, *p* = 0.04), moreover, subgroup analysis indicated that aquatic exercises were significantly associated with the effectiveness in the subgroup aged < 65 years and ≥ 65 years. Since only one article was included in the subgroup of age < 65 years (30), caution should be used in the interpretation and application of this finding. Most noteworthy, although no significant improvement was observed in ULS, LLS, agility, and flexibility in the subgroup aged ≥ 65years, aquatic exercise had positive effects on postmenopausal women of this age. It was concluded that aquatic exercise may be a better exercise method to improve the overall QoL of old postmenopausal women. Our findings are consistent with those of the study of Hafele et al. (39), which used aquatic aerobic exercise or a combination of aquatic aerobic and resistance exercise for 60 min three times a week for 16 weeks and showed significant improvements in overall QoL in both exercise intervention groups. However, there was no improvement in the control group (39). Similar to our study, Silva et al. (34) showed a significant 17% improvement in overall QoL in the aquatic aerobic exercise group adopting 12-week aquatic aerobic exercise or a combined aquatic aerobic and resistance exercise intervention with exercise twice a week. Although no beneficial effect of combined aquatic aerobic and resistance exercise was found (34), there was no denying of the relevance of aquatic exercises via improving postmenopausal women population in the area of the body (relating to the pain or discomfort, energy or fatigue, sleep, rest, mobility, daily activities, drug dependence and performance), psychology (including emotion, learning, memory and attention, self-esteem, appearance, spiritual, social, religious, and positive or negative thinking), social identity (personal relationship, social support and sex) and environment (including physical safety, home environment, financial security, information evaluating opportunities, participate in social or cultural activities and leisure time activities). This can be explained by previous studies, which showed that social and psychological problems such as depression, anxiety, or social isolation were significantly associated with chronic diseases (30, 96). In addition, subgroup analysis results showed that the resistance exercise and combination exercise subgroups had a significant improvement in overall QoL, while the aerobic exercise subgroup had no significant effect. The importance of resistance training for postmenopausal women was further emphasized here.

### Limitations

There are some limitations in the present study. First, few high-quality studies were included, and most were of moderate quality, especially in terms of blinding subjects, coaches, and measurements. Second, although more articles were included than the study of Saquetto et al. (36), there may still be the risk of insufficient literatures in the analysis of some indexes, especially in the subgroup analysis, so the results of the present study should be interpreted with caution when practicing.

### Implications

In the present systematic review and meta-analysis, we evaluated and updated the effects of aquatic exercise on physical fitness and quality of life (QoL) in postmenopausal women. The present findings indicate that, aquatic exercise significantly improved ULS, LLS, agility, flexibility, and overall QoL in postmenopausal women. Aquatic resistance exercise is recommended as the best option for postmenopausal women to improve physical fitness and QoL.

## Conclusions

Aquatic exercise significantly improved ULS, LLS, agility, flexibility, and overall QoL in postmenopausal women compared to those with no exercise. The beneficial efficacy of aquatic exercise on ULS, LLS, agility, and flexibility was only seen in postmenopausal women < 65 years old, but that on the overall QoL was seen both in postmenopausal women < 65 years old and ≥ 65 years old. Resistance exercise was better than aerobic/multicomponent exercise in the spectrum of aquatic exercise.

## Data availability statement

The original contributions presented in the study are included in the article/Supplementary material, further inquiries can be directed to the corresponding author.

## Author contributions

W-SZ conceptualized the study, searched the databases, extracted the data, performed the statistical analyses, and wrote an original draft. S-JM searched the databases, extracted the data, and reviewed, and edited the original draft. S-KZ evaluated the methodological quality and performed the statistical analyses. HX evaluated the methodological quality. W-LL edited the final draft. All the authors have reviewed and approved the final version of the manuscript.
