# Supplementary material for: Effects of aquatic exercises on physical fitness and quality of life in postmenopausal women: an updated systematic review and meta-analysis
Source: Front Public Health. 2023 Jun 8;11:1126126. doi: 10.3389/fpubh.2023.1126126 (PMC10285069; doi:10.3389/fpubh.2023.1126126)
Supplement: Supplementary file 1 [file Data_Sheet_1.pdf]

Supplementary Material

Effects of Aquatic Exercises on Physical Fitness and Quality of Life in Postmenopausal Women: An Updated Systematic Review and Meta-Analysis

Wen-Sheng Zhou[1*], Su-Jie Mao[2], Shi-Kun Zhang[3], Hong Xu[4], Wei-Lu Li[5]

*** Correspondence:** Wen-Sheng Zhou: zhouwensheng@njxzc.edu.cn

1 Supplementary Figures

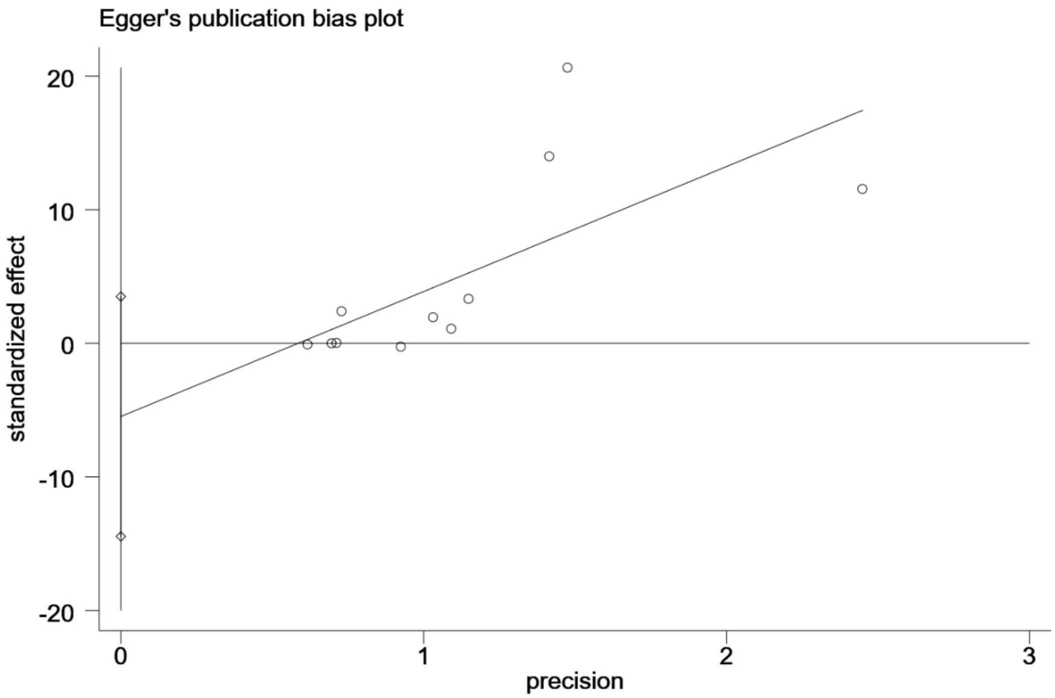

Supplementary Figure 1. Egger Plot of Lower Limbs Strength.

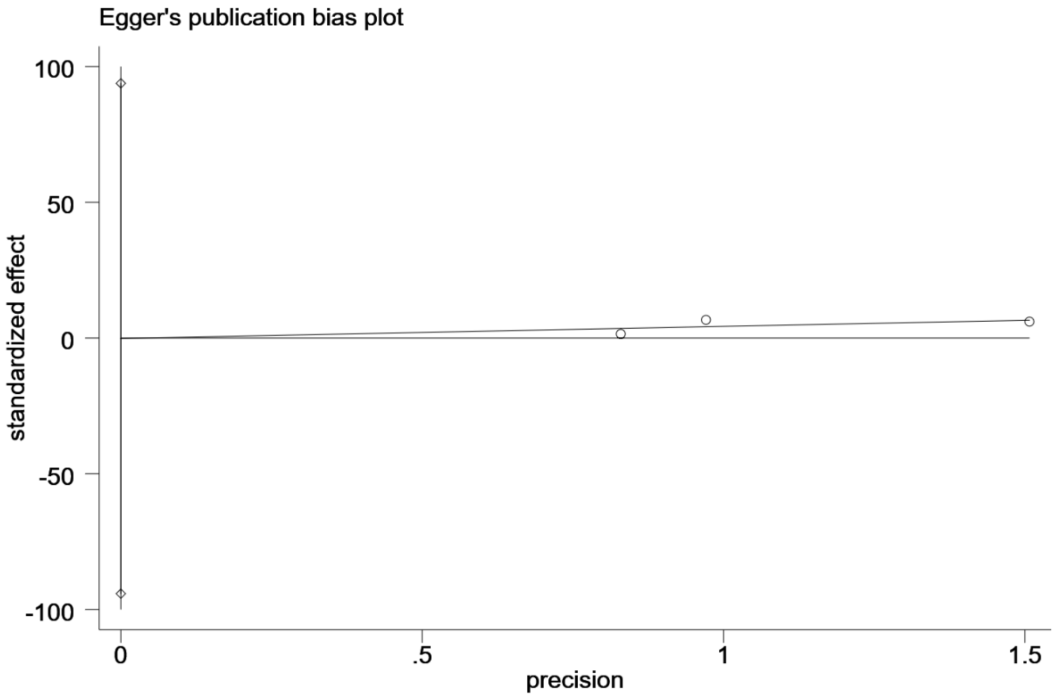

Supplementary Figure 2. Egger Plot of Upper Limbs Strength.

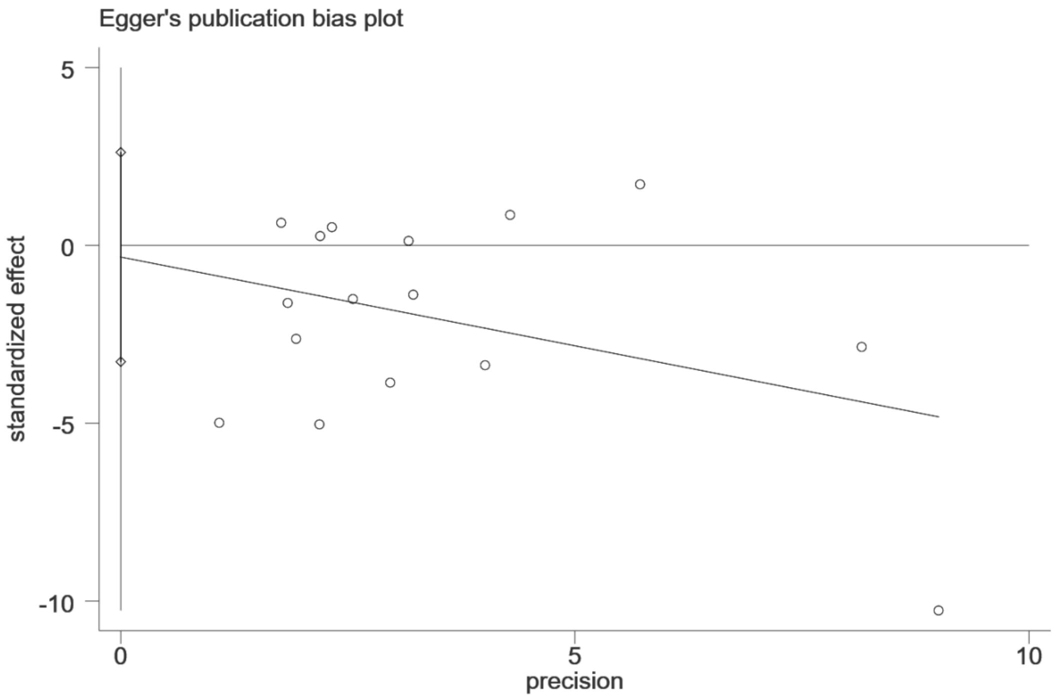

Supplementary Figure 3. Egger Plot of Agility.

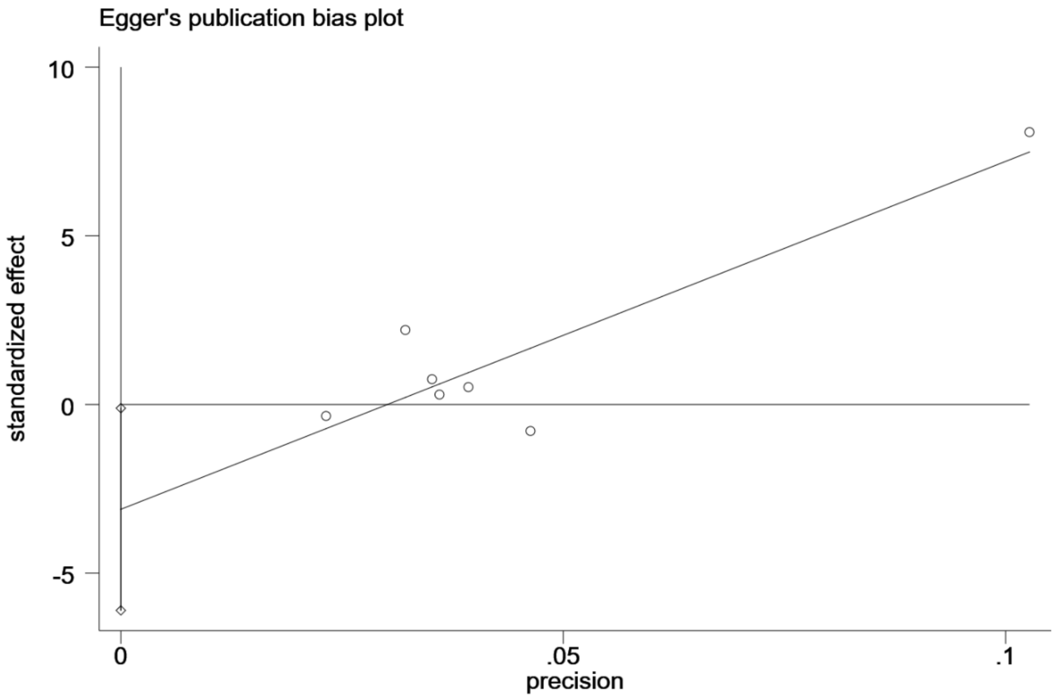

Supplementary Figure 4. Egger Plot of Aerobic Capacity.

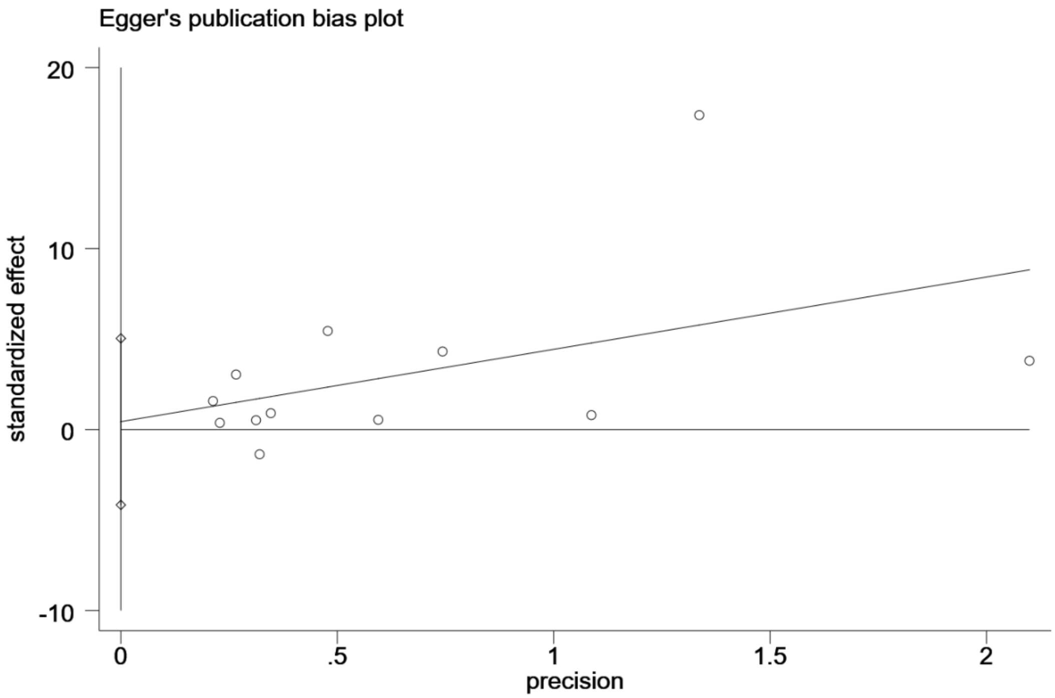

Supplementary Figure 5. Egger Plot of Flexibility.

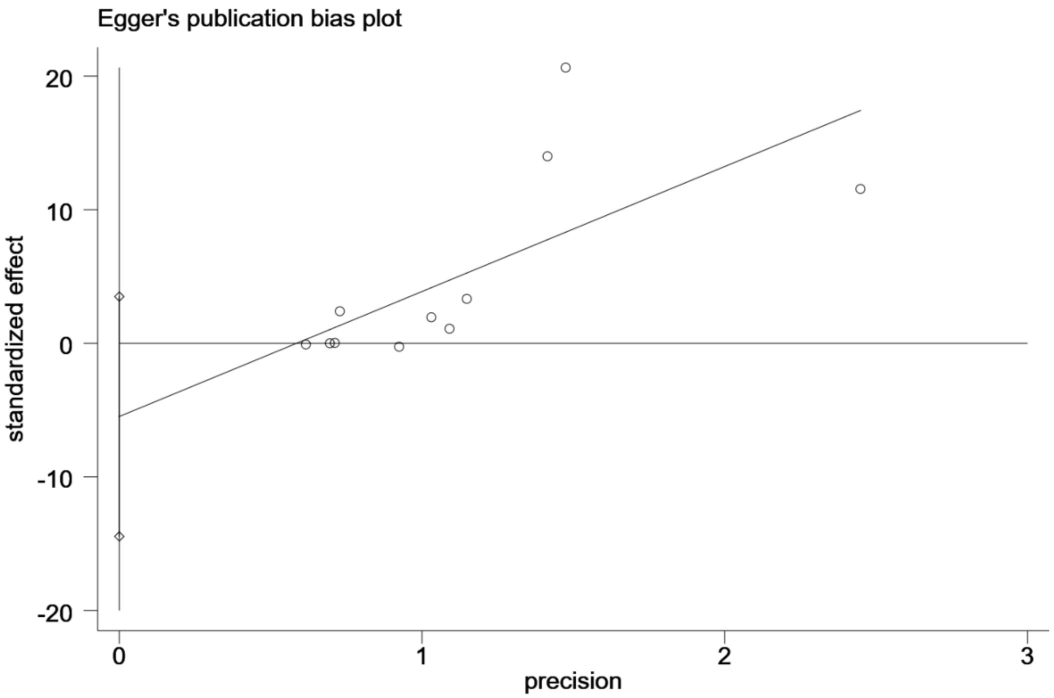

Supplementary Figure 6. Egger plot of Overall Quality of Life.
